# Activation of the Nrf2 Signaling Pathway as a Therapeutic Strategy Against Periodontal Disease: A Narrative Review

**DOI:** 10.3390/dj13070314

**Published:** 2025-07-11

**Authors:** Sarmistha Saha, Nadezhda Sachivkina, Ekaterina Lenchenko, Olga Pilshchikova, Alexandr Muraev

**Affiliations:** 1 Department of Biotechnology, Institute of Applied Sciences & Humanities, GLA University, Mathura 281406, Uttar Pradesh, India; 2 Department of Microbiology V.S. Kiktenko, Institute of Medicine, Peoples’ Friendship University of Russia Named After Patrice Lumumba (RUDN University), 117198 Moscow, Russia; 3 Department of Veterinary Medicine, Russian Biotechnological University (BIOTECH University), 125080 Moscow, Russia; lenchenko.ekaterina@yandex.ru; 4 Department of Therapeutic Dentistry, Institute of Medicine, Peoples Friendship University of Russia (RUDN University), 117198 Moscow, Russia; pilshchikova_ov@pfur.ru; 5 Department of Oral and Maxillofacial Surgery, Institute of Medicine, Peoples’ Friendship University of Russia Named After Patrice Lumumba (RUDN University), 117198 Moscow, Russia; muraev_aa@pfur.ru

**Keywords:** periodontitis, Nrf2, oxidative stress, pharmacotherapy

## Abstract

Periodontitis (PD), is a chronic inflammatory disease of the periodontal system, which includes gingiva, periodontal ligament, alveolar bone, and tooth cement. It is becoming increasingly prevalent globally, and its implications for oral health are profound. The Nrf2 signaling pathway is crucial in managing the relationship between inflammation and oxidative stress, making it vital for understanding this disease. Nrf2 interacts with key redox-sensitive inflammatory pathways, playing a vital role in the development of periodontitis. Acknowledging these dynamics underscores the importance of proactively addressing the complex aspects of periodontal disease. This review emphasizes its intricate interactions with redox-sensitive transcription factors vital for sustaining the self-perpetuating inflammatory processes underlying the disease. Additionally, it explores promising therapeutic strategies aimed at Nrf2 activation and encourages more effective management of PD.

## 1. Introduction

**Figure 1:**
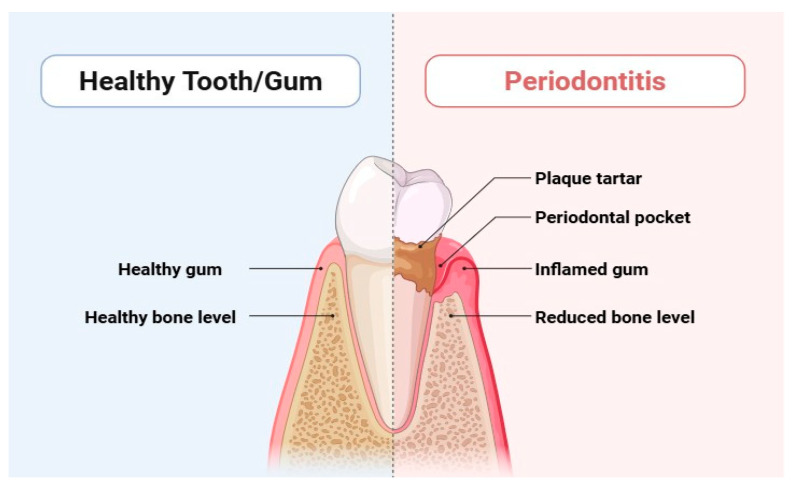
Differentiation between healthy tooth and periodontitis.

Periodontitis is a serious dysbiotic disease that causes chronic inflammation and tissue degradation, disrupting the delicate balance of microbial populations in the periodontal tissues [1,2]. This widespread inflammatory disease affects a staggering 10% to 15% of the population and can ultimately culminate in tooth loss [3], posing a significant threat to both systemic and oral health. Gingival bleeding, the formation of periodontal pockets, and the loss of periodontal tissue due to alveolar bone loss and clinical attachment loss are significant indicators of periodontitis (Figure 1) [4]. Alarmingly, periodontitis accounted for an overall incidence rate of 61.6% globally between 1990 and 2010, according to the Global Burden of Disease report. Recent updates indicate that its prevalence remains a pressing concern [4]. The consequences of periodontitis extend beyond oral health; it contributes to masticatory dysfunction and edentulism, which in turn negatively impacts overall health and increases the financial burden of dental treatments [5]. Individuals may experience tooth loss, instability, impaired chewing ability, altered appearance, socioeconomic disparities, and diminished quality of life [5].

The most severe cases of periodontitis are classified as stages III and IV. These stages are characterized by angular irregularities, furcation involvements, dental mobility, and impairment of function. During these advanced stages, many intrinsic and environmental risk factors hinder the host’s ability to combat bacterial infections and manage tissue damage. Unfortunately, while only 10–12% of individuals suffer from severe periodontitis, it often afflicts several teeth per person [6,7]. This interaction occurs when various bacterial infections in dental plaque lead host cells to produce cytokines that promote inflammation. This immune response generates an oxidative burst, resulting in proteolytic enzymes and reactive oxygen species (ROS) generation [8].

The most common bacterial offenders associated with periodontitis include *Aggregatibacter actinomycetemcomitans* (*A. actinomycetemcomitans*), *Porphyromonas gingivalis* (*P. gingivalis*), and *Tannerella forsythia* (*T. forsythia*) [9]. These pathogens possess various virulence factors, such as fimbriae, adhesins, lipopolysaccharides, hemagglutinins, proteinases, and toxic metabolites, that enable their survival and proliferation [10]. Recognizing and addressing the gravity of periodontitis is crucial for safeguarding individual health and well-being. Periodontal bacteria are armed with potent virulence factors that not only damage host tissues but also undermine the immune response, making them a formidable threat. These factors include proteases, lipopolysaccharides (LPS), and other enzymes [11]. LPS is particularly notable for its ability to trigger the production of proinflammatory cytokines, thereby heightening the immune response while simultaneously being highly immunogenic. Proteases exacerbate this issue by degrading crucial extracellular matrix components and host immune proteins [12]. Furthermore, the development of periodontitis is influenced not only by these bacterial factors but also by host-related factors. Genetic predisposition and systemic disorders, such as diabetes, compound the risk and further complicate the pathogenesis [13]. An insightful observational study was undertaken to explore the critical connection between aortic valve replacement patients and periodontal disease. This research not only assessed the presence of oral pathogens in aortic valve specimens but also contrasted these microorganisms with those identified in the oral cavity. Understanding these relationships could have significant implications for patient care and highlight the importance of oral health in preventing cardiovascular conditions. The comparison of microorganism profiles in the heart and mouth reveals critical insights into their interconnectedness. The presence of these microorganisms is closely associated with a direct mechanism, driven by the inflammation characteristic of periodontal pockets that enlarge the vascular bed. This connection is underscored by the alarming finding that similar oral bacterial DNA species were significantly present in cardiac samples from patients with periodontitis, unlike those from edentulous patients [10]. This evidence highlights the potential implications of oral health on cardiovascular wellness, urging us to reconsider the importance of maintaining good periodontal health.

The nuclear factor erythroid 2-related factor 2 (NFE2L2, or Nrf2) works in collaboration with Kelch-like ECH Associated Protein 1 (Keap1) for providing protection against oxidative stress [14]. This important pathway plays a crucial role in reducing reactive oxygen species (ROS) by activating a variety of genes that produce powerful antioxidants and phase II detoxifying enzymes. In its inactive state, Nrf2 is located in the cytoplasm, where it is tightly bound to Keap1. This interaction is essential, as Keap1 is a key component of the Cullin 3 (Cul3)/RING box protein 1 (RBX1) E3 ubiquitin ligase complex, which regulates Nrf2 activity [14]. This complex facilitates the ubiquitination and degradation of Nrf2, preventing it from exerting its protective effects. Recognizing and harnessing this pathway could be transformative for enhancing cellular health and combating oxidative damage. Keap1 harbors cysteine residues that serve as vigilant sensors of the intracellular redox environment. When these cysteine residues undergo oxidation in response to oxidant stimuli, Keap1 undergoes transformative conformational changes. This process effectively halts the proteasomal degradation of Nrf2, allowing it to migrate into the nucleus where it can exert its protective influence. Nrf2 plays a crucial role in regulating the expression of antioxidant enzyme genes within the nucleus. It achieves this by binding to antioxidant response elements (AREs) that are present in the promoters of these genes, thereby initiating their transcription [15].

As highlighted by Hatipoglu et al. [16], periodontitis is closely linked to the activation of polymorphonuclear leukocytes, which unleash reactive oxygen species (ROS) during inflammatory episodes. This situation leads to oxidative stress, a critical condition marked by an excess of ROS and oxidative damage [17]. Understanding these dynamics reinforces the urgent need to address the multifaceted nature of periodontal disease, emphasizing the vital intersection between bacterial virulence factors and host vulnerabilities.

## 2. Interplay of Nrf2 Signaling Pathway and Oxidative Stress in Periodontitis

Research highlights the important role that neutrophils play in the development of periodontitis, as they serve as the primary source of reactive ROS [18]. Neutrophils utilize the NADPH oxidase pathway to generate an abundance of ROS as they effectively phagocytose periodontal pathobionts [19]. Recent studies showed that peripheral blood neutrophils from people with chronic periodontitis produce more ROS when exposed to purified immunoglobulin-opsonized *Staphylococcus aureus*, compared to those from healthy individuals. This finding strongly indicates the role of the Fc-gamma receptor (FcγR) pathway in activating the hyper-reactive neutrophil phenotype commonly observed in periodontitis patients [20,21]. A study by Fredriksson et al. highlights that neutrophils in these individuals are more likely to activate the FcγR pathway [22]. This finding contrasts with the activation of the complement receptor CR3 or the intracellular protein kinase C enzyme [22]. Importantly, periodontal therapy has shown promise in diminishing the ROS production triggered by the FcγR pathway, whether or not there is prior sensitization with *P. gingivalis* and *F. nucleatum*. However, it is crucial to note that this treatment does not significantly impact the levels of unstimulated extracellular ROS [23]. Moreover, the same research underscores that the hyperreactivity of neutrophils in periodontitis arises from both intrinsic and reactive mechanisms [23]. Recent in vitro studies have revealed that periodontal pathogens and their components play a significant role in activating not only neutrophils but also a variety of phagocytes and important cells within periodontal tissues, including monocytes, gingival fibroblasts, and periodontal ligament cells. This activation leads to the production of heightened levels of ROS [24,25]. Yet, to fully understand the implications of this process on oxidative stress in periodontitis, further research is essential.

Monitoring changes in oxidative stress biomarker levels offers valuable insights into the progression of periodontitis. Moreover, a wealth of research has shown that periodontitis is associated with diminished activity of crucial enzyme antioxidants, including catalase (CAT) and superoxide dismutase (SOD) [26]. The results highlight key differences in oxidative stress biomarker levels between individuals with periodontitis and healthy controls. These biomarkers encompass malondialdehyde levels, glutathione peroxidase, nitric oxide, total oxidative status, and 8-hydroxydeoxyguanosine. Notably, patients with periodontitis exhibit increased indicators of ROS-induced tissue damage found in their gingival fluid and inflamed periodontal tissue [27].

Recent studies have illuminated critical issues, such as the apoptosis of human periodontal ligament stem cells (hPDLSCs), and the alarming loss of alveolar bone [19]. It is important to recognize that ROS, when maintained at the right levels, is indispensable for vital biological processes like wound healing, apoptosis, migration, and cell proliferation [28]. A key player in this context is dynamin-related protein 1 (Drp1) and ROS can enhance the expression of Drp1, yet this can lead to detrimental effects, such as mitochondrial dysfunction manifesting as reduced ATP levels and abnormal mitochondrial membrane potential, ultimately triggering hPDLSC apoptosis [19]. On a more positive note, low concentrations of ROS can encourage the differentiation and proliferation of PDLFs in culture, highlighting their dual roles. However, we must note that excessive ROS can inflict cytotoxic damage on periodontal tissue [29].

**Figure 2:**
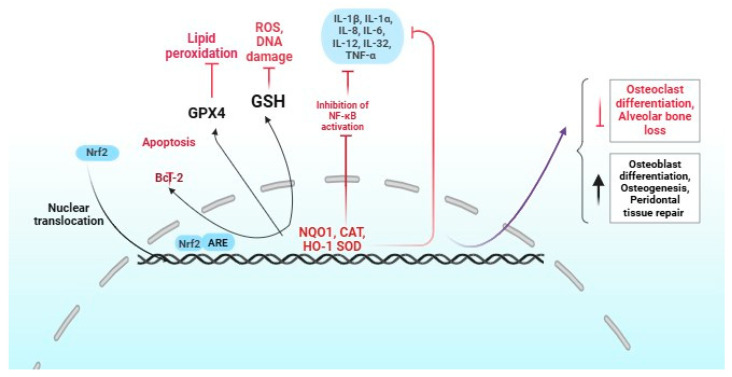
Role of Nrf2 activation in periodontitis. The red and black colors indicate positive and negative regulation by Nrf2, respectively.

An important strategy for preventing periodontal disease involves effectively managing oxidative stress in healthy periodontal tissue. This highlights the crucial role of Nrf2 in protecting these tissues, even in the face of continuous interactions with bacteria, neutrophils, and macrophages (Figure 2) [30].

The Nrf2 pathway plays a crucial role in combating severe periodontitis, a condition that leads to heightened levels of polymorphonuclear neutrophils (PMNs) and significant oxidative damage. Research demonstrates that mice deficient in Nrf2 (Nrf2^−/−^) not only exhibit reduced catalase production by PMNs but also experience increased destruction of periodontal tissue [31,32]. Moreover, the absence of Nrf2 plays a critical role in the activation of RANKL-induced kinases and nuclear factor-activated T cells. This process promotes osteoclast differentiation and ultimately contributes to the loss of alveolar bone [32]. In experimental models of periodontitis, nuclear Nrf2 showed elevation, alongside elevated expression of heme oxygenase-1 and heightened luciferase activity compared to healthy controls [33]. Overexpressing Nrf2 significantly elevates the production of critical antioxidants, such as NADPH-quinone oxidoreductase 1, heme oxygenase, and γ-glutamylcysteine synthetase, facilitating cell proliferation and curtailing apoptosis [34]. This highlights the essential function of Nrf2 in preserving the antioxidant capacity of hPDLSC. Despite the challenges, there remains an urgent need to unravel the intricate web of oxidative damage and antioxidant mechanisms that underpin periodontal tissues. It has been shown that Nrf2 directly inhibits the NLRP3-associated genes [35], indicating that Nrf2 may be a therapeutic inhibitor of periodontitis. 

A number of evidence highlights the concerning link between increased oxidative stress and peri-implant disease, characterized by higher levels of myeloperoxidase and changes in nitric oxide metabolism [36,37]. This pivotal research suggests a notable rise in Parkinsonism-associated deglycase 7, directly involved in the Nrf2 pathway regulation, alongside elevated levels of 8-hydroxy-deoxyguanosine. However, despite these findings, there is no change in Nrf2 expression in the mucosa of peri-implantitis patients [38]. This gap in understanding calls for further investigation and awareness of the oxidative stress mechanisms at play in dental peri-implant disease.

Some studies also highlight the crucial role of ROS in driving osteoclastogenesis [39,40]. Remarkably, osteoclastic bone resorption is significantly heightened in Nrf2-deficient osteoclast precursor cells compared to their wild-type counterparts [41]. Moreover, utilizing Keap1 luciferase reporter mice provides valuable insights into the enhanced nuclear localization of Nrf2 within inflammatory periodontal tissues, which further underscores the protective function of Nrf2 in maintaining periodontal health [33].

## 3. Pharmacotherapy of Periodontitis via Nrf2 Signaling Pathway

The treatment strategies of periodontitis presents significant challenges due to the complexities of the condition, the complications that often arise after antimicrobial treatment, and our limited understanding of the interactions among various bacterial species involved in the disease [42]. Antibiotic-resistant bacterial biofilms present a challenge in the healing process of soft tissues. However, understanding that both the host’s immune response and the bacterial load play a crucial role in the severity of the condition opens the door to potential solutions [43]. In this regard, natural products offer a promising approach to treating oral infections. They work by effectively limiting microbial adhesion, which helps reduce the overall bacterial count through their bactericidal properties [44]. Additionally, these products can block the formation of water-soluble glucan, inhibit amylases, and disrupt biofilm formation.

In a very recent study, Nootkatone, a sesquiterpenoid, demonstrates remarkable efficacy in combating alveolar bone loss and inflammation by engaging the Nrf2/HO-1 pathway [45]. In rats with induced periodontitis, treatment with nootkatone significantly reduced the levels of inflammatory markers, such as IL-1β, IL-6, and TNF-α within the gingival tissue, showcasing its potential to protect against the degradation of alveolar bone. This enhancement of antioxidant activity resulted in a marked decrease in oxidative stress, evidenced by heightened SOD levels and reduced MDA concentrations. The findings underscore the transformative potential of nootkatone in periodontal health, positioning it as a promising therapeutic agent in the fight against periodontitis.

Another study has highlighted the benefits of quercetin treatment for hPDLCs exposed to hydrogen peroxide. Quercetin significantly elevates the expression of key protective proteins, including Nrf2, NQO1, CAT, and HO-1. Notably, it also promotes osteogenesis in hPDLCs under oxidative stress. Furthermore, in animal models of periodontitis, quercetin enhances the expression of Nrf2 and SOD, while at the same time minimizing alveolar bone loss [46]. However, the precise mechanisms by which quercetin interacts with Nrf2 signaling are still not fully understood. Therefore, it is crucial to conduct further research aimed at identifying the most effective dosage and delivery method. This is especially important given the challenges posed by its low bioavailability, limited water solubility, and the inactivity of its metabolites.

A recent study has shown that curcumin significantly enhances the osteogenic differentiation of hPDLSCs by activating the PI3K/AKT/Nrf2 signaling pathway. This activation increases AKT phosphorylation and Nrf2 expression, allowing Nrf2 to enter the nucleus and exert its effects. Notably, inhibiting the PI3K/AKT pathway with a specific inhibitor LY294002 leads to a significant decrease in Nrf2 expression. Additionally, silencing Nrf2 with siRNA reverses the osteogenic differentiation prompted by curcumin in hPDLSCs [47]. This shows curcumin’s potential as a modulator of osteogenesis through the PI3K/AKT/Nrf2 pathway. Curcumin’s potential to stimulate osteogenesis via the PI3K/AKT axis remains unclear, though. Moreover, curcumin’s efficacy extends to oral health, as shown by its pretreatment of an H400 cells exposed to *F. nucleatum*, which showed enhancement of the expression of Nrf2 and HO-1 [48].

Li et al. showed that the natural phenolic compound paeonol, significantly reduced RANKL expression and effectively inhibited the osteoclast formation in periodontitis-induced rats [49]. Moreover, paeonol has been shown to effectively inhibit NF-κB activation and reduce oxidative stress. This is achieved by enhancing HO-1 expression and increasing GSH levels in gingival tissues. Most impressively, paeonol was shown to enhance Nrf2 expression, while silencing Nrf2 diminished its protective effects on NF-κB activation [49].

Another study found that administering resveratrol to rats suffering from periodontitis not only alleviated alveolar bone resorption but also activated vital pathways, including sirtuin 1 (SIRT1)/AMP-activated protein kinase (AMPK) and Nrf2, within inflamed gingival tissues. Furthermore, resveratrol significantly lowered the levels of pro-inflammatory markers, such as TNF-α, IL-1, and IL-6 [50]. However, the authors did not assess the levels of resveratrol in serum or gingival tissue.

In primary neutrophils derived from periodontitis and healthy controls, the natural compound sulforaphane (SFN) has shown promising effects. It not only increased intracellular levels but also decreased the ratio of GSH to GSSG and reduced the neutrophil respiratory burst [51]. Moreover, SFN has been found to decrease harmful extracellular oxygen radical generation while simultaneously increasing the expression of key protective proteins, such as Nrf2, NQO1, and glutamate cysteine ligase catalytic and modifier subunits (GCLC and GCLM) [52]. The benefits of SFN extend to gingival epithelial cells as well, where treatment with SFN leads to substantial increases in the Nrf2 expression [53]. Emerging evidence indicates that hesperetin could also be a therapeutic option for periodontitis. This compound effectively inhibits RANKL-induced osteoclastogenesis, curtails osteoclastic bone resorption, and significantly disrupts the activation of NF-κB and MAPK signaling pathways in RAW 264. Furthermore, hesperetin increases the Nrf2, HO-1, and NQO1expression levels, thereby enhancing the body’s ability to scavenge harmful ROS [54].

Zhang et al. [55] conducted a study on rats with experimental periodontitis and found that Biochanin A treatment significantly reduced alveolar bone resorption and levels of inflammatory markers, such as IL-1β, TNF-α, and ROS. Biochanin A treatment enhances Nrf2 protein expression, indicating its potential to inhibit inflammation and prevent bone loss in periodontitis. Likewise, studies show that 10-oxo-trans-11-octadecenoic acid (KetoC) significantly increases the expression of Nrf2, HO-1, and NQO1 in gingival epithelial cells. This remarkable effect leads to a reduction in ROS levels [56].

Research reveals that when primary human gingival fibroblasts are treated with caffeic acid phenethyl ester (CAPE), HO-1 expression was enhanced significantly [57]. Furthermore, when Nrf2 is silenced, CAPE’s positive effects on HO-1 expression in macrophages are reduced. CAPE significantly lowers pro-inflammatory cytokines IL-1α and IL-1β in response to periodontal pathogens. Additionally, inhibiting HO-1 with SnPP diminishes both the antioxidant and anti-inflammatory effects of CAPE, emphasizing the importance of HO-1 in these processes.

Euphorbia factor L1 (EFL1), a diterpenoid, has demonstrated significant efficacy in combatting osteoclast formation and bone resorption. In studies utilizing mouse bone marrow-derived macrophages, EFL1 effectively inhibits RANKL-induced c-Fos expression, showcasing its ability to disrupt harmful bone degradation pathways [58]. Moreover, EFL1’s impact extends to reducing ROS, thereby activating Nrf2 signaling pathways that enhances the protective molecules, such as sulfiredoxin (SRX), peroxiredoxins (PRXs), and thioredoxins (TRXs). This multifaceted mechanism not only induces apoptosis in mature osteoclasts but also curtails inflammation-induced bone erosion. Dehydrocostus lactone (DL) is a promising natural sesquiterpene lactone that significantly reduces NF-κB activation in RANKL-stimulated RAW 264.7 cells. This promising intervention also elevated the expression of key protective proteins, including Nrf2, NQO1, and PRX1, all while effectively lowering ROS levels. Strikingly, the silencing of Nrf2 led to an increase in osteoclast differentiation [59].

Notopterol, a furanocoumarin, shows great promise in combating periodontitis. Studies indicate that treatment with notopterol significantly lowers levels of pro-inflammatory cytokines in LPS-stimulated human gingival fibroblasts. This remarkable effect is achieved by inhibiting the NF-κB signaling pathway, specifically by blocking the p65 subunit phosphorylation, a key player in inflammation [60]. Notopterol also elevates the phosphorylation of AKT and PI3K and Nrf2 expression. Interestingly, the effectiveness of notopterol is reduced when the AKT inhibitor MK-2206 is introduced. In essence, notopterol alleviates periodontal inflammation, functioning through dual mechanisms: it inhibits the activation of the NF-κB pathway while simultaneously harnessing the antioxidant potential of Nrf2 and the PI3K/AKT signaling pathways [61].

Isorhamnetin, a potent flavonoid, has demonstrated remarkable anti-inflammatory properties by effectively reducing the release of nitric oxide, prostaglandin E2 and inflammatory cytokines in human gingival fibroblasts exposed to LPS [62]. This compound not only inhibits the NF-κB activation by blocking the phosphorylation of its p65 subunit, but it also elevates the levels of Nrf2 and HO-1. Furthermore, when Nrf2 is silenced, HO-1 expression diminishes, leading to the unchecked activation of NF-κB and a subsequent surge in inflammation. In addition, another phytochemical, magnolol has shown significant promise in combating inflammation caused by LPS from *P. gingivalis* in macrophages [63]. Intriguingly, when p38 MAPK activity is inhibited, the activation of the Nrf2/HO-1 pathway by magnolol is also reduced. Additionally, the reverse effect of the SnPP inhibitor on HO-1 activity corroborates the conclusion that magnolol’s anti-inflammatory action is largely dependent on activating the Nrf2/HO-1 axis [64]. These findings not only affirm the effectiveness of magnolol in reducing inflammation but also present a therapeutic agent in the treatment of periodontitis.

Recent research reveals its potent effect of schisandrin on macrophages stimulated by LPS from *P. gingivalis*. Notably, schisandrin effectively reduces the secretion of key pro-inflammatory cytokines, such as TNF-α, IL-1β, and IL-6, while also inhibiting the activation of NF-κB by targeting the expression of its p65 subunit [65]. Furthermore, schisandrin promotes HO-1 expression by enhancing Nrf2 levels and activating the PI3K/Akt and ERK pathways. Sappanchalcone, another natural flavonoid, has also shown to elevate significantly the HO-1 expression in human dental pulp cells (HDPCs) and hPDLCs. It also protects from cytotoxicity and oxidative stress induced by H_2_O_2_ [66]. Moreover, it effectively inhibits the LPS-stimulated release of nitric oxide, PGE2, and various interleukins. The enhancement in HO-1 levels is linked to the Nrf2 and c-Jun NH2-terminal kinase (JNK) upregulation, further emphasizing the multifaceted benefits of sappanchalcone. Astaxanthin, a potent carotenoid, has demonstrated impressive protective effects against oxidative stress and growth inhibition in hPDLCs induced by advanced glycation end products [67]. By activating the Nrf2 pathway, astaxanthin combats these detrimental processes. It effectively reduces oxidative stress and inflammatory responses while inhibiting osteoclastic activity. These findings indicate that, although astaxanthin may not directly lower high blood sugar levels, it plays a crucial role in alleviating periodontal destruction and addressing systemic oxidative complications associated with type 1 diabetes.

Adjuvant antioxidant therapy presents an innovative approach for effectively managing periodontitis [68]. While the use of antioxidant enzymes like SOD has been explored, research in osteoarthritis reveals that simple intra-articular injections of SOD fall short of the potential offered by activating Nrf2 to combat oxidative stress and its associated pathological conditions [69]. This insight is particularly relevant for periodontitis because Nrf2 activation influences the expression of a vast array of antioxidant gene products essential for defending against damaging oxidants and free radicals. By harnessing the power of Nrf2 through systemic activation, we have the opportunity to significantly inhibit tissue destruction associated with periodontitis [51]. Embracing this strategy could revolutionize our approach to treating periodontitis and improve patient outcomes dramatically.

For instance, previous studies have demonstrated the central role of Nrf2 in systemic inflammatory diseases characterized by chronic pain, such as fibromyalgia [70]. At the same time, the bidirectional relationship between periodontitis and conditions including fibromyalgia [71] and chronic pain [72] has been proposed. As such, further studies are warranted to explore whether the Nrf2 pathway plays a role in the connection between periodontitis and these conditions. Likewise, given that the Nrf2 pathway is involved in the pathogenesis of Alzheimer’s disease [73], and that periodontitis is commonly observed among patients with dementia, including Alzheimer’s disease [74], it is clinically relevant to investigate whether targeting the Nrf2 pathway could mitigate the link between periodontitis and dementia. A very recent study has revealed that adhesive, mineralized hydrogel microspheres infused with the traditional phytocompound cordycepin (MCD) have the remarkable ability to rejuvenate impaired PDLSCs that suffer from inflammation-induced suppression [75]. This innovative approach addresses the critical challenge of restoring osteogenesis and ligament formation. Mechanistically, MCD effectively combats premature cell aging triggered by inflammation via the Nrf2 pathway, while simultaneously minimizing DNA damage. In in vivo experiments using a rat model of periodontitis, MCD demonstrated significant potential in enhancing periodontal bone regeneration by not only stimulating osteogenesis but also by curbing osteoclastic activity. Treatment with four-octyl itaconate proves to be a potent approach to combat inflammation triggered by LPS in the periodontal microenvironment. Not only does 4-OI significantly reduce inflammation, but it also plays a vital role in preventing alveolar bone loss through the activation of Nrf2 [76]. Further validation of 4-OI’s properties emerged when studies showed that silencing Nrf2 effectively negated the antioxidant benefits of 4-OI by downregulating key antioxidant enzymes. Additionally, the role of Nrf2 becomes evident in experiments involving Nrf2^−/−^ mice, where 4-OI treatment failed to protect against alveolar bone dysfunction due to induced periodontitis. However, to validate the effectiveness of 4-OI, it is essential to conduct additional clinical trials involving larger participant groups and prolonged observation periods.

Another study successfully synthesized melatonin-derived carbon dots (MTCDs) which showed a crucial role in regulating intracellular ROS levels, thereby maintaining mitochondrial homeostasis and effectively suppressing the production of inflammatory mediators [77]. A recent interesting study has introduced the innovative TM/BHT/CuTA hydrogel system, ingeniously crafted through the self-assembly of a copper-based nanozyme, specifically, copper tannic acid coordination nanosheets, and a triglycerol monostearate/2,6-di-tert-butyl-4-methylphenol (TM/BHT) hydrogel [78]. This hydrogel stands out with its ability to maintain a presence at inflammation sites, owing to its negatively charged composition that attracts and binds positively charged elements through electrostatic adsorption. As periodontitis progresses and matrix metalloproteinases (MMP) levels rise, the hydrogel hydrolyzes, enabling the precise release of the CuTA nanozyme. Also, this nanozyme is capable of influencing macrophage behavior, shifting from the pro-inflammatory M1 phenotype to the healing M2 phenotype through the Nrf2/NF-κB pathway. This shift results in a significant reduction in pro-inflammatory cytokines, and the promotion of osteogenic gene expression, all of which contribute to reduced inflammation and accelerated tissue regeneration in cases of periodontitis. A novel type of N-acetyl-l-cysteine-derived red fluorescent carbonized polymer dot (NCPD) showed to serve as a potent extracellular antioxidant, effectively neutralizing ROS and protecting cellular health [79]. NCPDs significantly enhance osteogenic differentiation in hPDLCs under H_2_O_2_ stress. Furthermore, these particles selectively accumulate in alveolar bone in vivo, leading to a substantial reduction in alveolar bone resorption.

In most recent research, the application of Ginsenoside Rg1 demonstrates promising effects in reducing the secretion of inflammatory factors, specifically IL-6, while also promoting an increase in the secretion of the TGF-β [80]. Furthermore, Ginsenoside Rg1 appears to effectively decrease the number of osteoclasts and enhances the expression of critical proteins, such as RUNX2 and OCN. In addition, Ginsenoside Rg1 improves the expression of Nrf2, while lowering the expression of Keap1. These findings indicate that Ginsenoside Rg1 may serve as an effective means to alleviate periodontitis via Keap1/Nrf2 pathway. However, cellular research is necessary to uncover the deeper mechanisms behind Rg1-regulated periodontitis. As a result, more research is required to ascertain how Rg1 functions in cells linked with periodontal tissue.

## 4. Conclusions

Periodontitis is a significant microbial disease that triggers an inflammatory response from the host, leading to the activation of specific proteinases. The periodontal ligament fibers break down as a result of this process, the junctional epithelium migrates apically, and harmful bacterial biofilm spreads across the root surface. Understanding the roles of specific proteins and their interactions with key signaling pathways, such as Nrf2, is paramount. In this review, we have examined a range of studies that shed light on the intricate roles this pathway plays in vital processes, including osteogenesis and inflammation. This insight can pave the way for innovative, patient-specific therapeutic strategies. Notably, Nrf2 stands out for its antioxidant and anti-inflammatory properties, acting as a crucial regulator of cytoprotective genes that safeguard tissues from damage. Furthermore, advances in research on inflammasome regulation, coupled with the right drugs, hold the promise of personalized treatments that can guide dental professionals in delivering tailored care. Given that Nrf2 activation plays an essential role in shielding cells from oxidative stress and inflammation, as well as in staving off alveolar bone loss, the development of specialized drugs or dietary supplements derived from natural compounds with these protective qualities could have profound implications not only for the treatment of periodontitis but also for the effective prevention of this pervasive disease. In this regard, future research should aim to explore the Nrf2/Keap1 signaling pathway, as it presents a valuable opportunity to enhance various cellular functions.

## Data Availability

No new data were created or analyzed in this study. Data sharing is not applicable to this article.

## Abbreviations

The following abbreviations are used in this manuscript:

LPS	lipopolysaccharides
ROS	reactive oxygen species
Nrf2	nuclear factor erythroid 2-related factor 2
Keap1	Kelch-like ECH Associated Protein 1
RBX1	RING box protein 1
FcγR	Fc-gamma receptor
CAT	catalase
SOD	superoxide dismutase
MDA	malondialdehyde
hPDLSCs	human periodontal ligament stem cells
Drp1	dynamin-related protein 1
PMNs	polymorphonuclear neutrophils
HO-1	heme oxygenase-1
NF-κB	nuclear Factor-κB
NQO1	NADPH:quinone oxidoreductase 1
GSH	glutathione
